# Normal T Cell Selection Occurs in CD205-Deficient Thymic Microenvironments

**DOI:** 10.1371/journal.pone.0053416

**Published:** 2012-12-31

**Authors:** William E. Jenkinson, Kyoko Nakamura, Andrea J. White, Eric J. Jenkinson, Graham Anderson

**Affiliations:** Medical Research Council Centre for Immune Regulation, University of Birmingham, Birmingham, United Kingdom; Institut Pasteur, France

## Abstract

The thymus imparts a developmental imprint upon T cells, screening beneficial and self-tolerant T cell receptor (TCR) specificities. Cortical thymic epithelial cells (CTEC) present self-peptide self-MHC complexes to thymocytes, positively selecting those with functional TCRs. Importantly, CTEC generate diverse self-peptides through highly specific peptide processing. The array of peptides utilized for positive selection appears to play a key role in shaping TCR repertoire and influencing T cell functionality. Whilst self-peptide diversity influences T cell development, the precise source of proteins generating such self-peptide arrays remains unknown, the abundance of apoptotic thymocytes failing thymic selection may provide such a pool of self-proteins. In relation to this notion, whilst it has been previously demonstrated that CTEC expression of the endocytic receptor CD205 facilitates binding and uptake of apoptotic thymocytes, the possible role of CD205 during intrathymic T cell development has not been studied. Here, we directly address the role of CD205 in normal thymocyte development and selection. Through analysis of both polyclonal and monoclonal transgenic TCR T-cell development in the context of CD205 deficiency, we demonstrate that CD205 does not play an overt role in T cell development or selection.

## Introduction

Conventional αβ T cell development occurs within the anatomically and functionally unique microenvironment of the thymus. Cortical and medullary regions constitute the main anatomical divisions of the thymus, being primarily defined by highly specialized cortical thymic epithelial cells (CTEC) and medullary thymic epithelial cells (MTEC). Functionally, CTEC mediate positive selection of CD4^+^8^+^ thymocytes expressing αβTCRs with low affinity for self-peptide/MHC complexes, whilst MTEC are specialized for negative selection and removal of auto-reactive T cell clones expressing high affinity αβTCRs for self-antigens, in addition to supporting the development of natural Foxp3^+^ regulatory T cells.

Although the precise mechanisms by which CTEC efficiently mediate positive selection remain unclear, several studies have shown CTEC possess specific intra-cellular machinery contributing to the generation of self-peptides, in addition to the constitutive expression of both MHC class I and II molecules. Amongst recently discovered peptide processing machinery expressed by CTEC, molecules such as the thymoproteasomal subunit β5t, the Thymus-specific serine protease (Tssp), and the cysteine protease Cathepsin-L play central roles in the generation of peptides required to select functionally diverse CD4 and CD8 T cells [1]–[3]. Importantly, absence of such CTEC specific peptide-processing elements alters selection of both polyclonal and transgenic monoclonal TCR specificities possibly occurring due to shifts in the array of peptides presented by CTEC.

While the mechanisms by which self-peptides contribute to the diversity of selected TCR specificities remains unclear, evidence suggests that a variety of different peptide fragments are required to ensure normal selection of diverse αβTCR repertoires [2]. Further, the precise cellular origin of self-peptide generating proteins, and the processes that enable self-proteins to enter antigen processing/presentation pathways in CTEC, is incompletely understood. Interestingly, experimental data has indicated that constitutive autophagy operates within CTEC. Of particular note, previous studies have demonstrated that in the absence of CTEC autophagy, selection of T cells was perturbed, suggesting that CTEC intrinsic pathways provided an essential source of material for self-peptide generation [4]. In contrast, a second study has argued that specific ablation of autophagy pathways within CTEC does not lead to significant alterations within thymocyte selection [5]. Together, such data suggest that alternative routes may exist for the generation of selecting self-peptides.

CD205 represents a C-type lectin placed within the Mannose receptor family [6]. In contrast to the Mannose Receptor, CD205 does not exhibit properties of lectin-binding and the full definition of physiological ligands bound by CD205 have yet to be revealed. Studies investigating the functional role of CD205 have predominantly focused on CD205 expression by peripheral dendritic cell subsets. Interestingly, such experiments have demonstrated that CD205 operates as a functional endocytic receptor [6]. Importantly, targeting of protein-coupled monoclonal antibody to CD205 in vivo leads to dendritic cell internalization of protein, peptide processing and subsequent presentation to T cells [7], [8]. CD205-mediated endocytosis leads not only to presentation on MHC class II but also MHC class I molecules via cross-presentation mechanisms facilitating recognition of exogenously derived peptides by both CD4 and CD8 T cells [7]. Interestingly, CD205 is expressed by cortical but not medullary thymic epithelium [6], raising the possibility that CD205 expression by CTEC may be involved in the uptake of self proteins and the generation of self-peptides for thymocyte positive selection. Previous in vitro studies using thymic epithelial cell lines have suggested that CD205 expressed by CTEC both binds, and facilitates, uptake of apoptotic and necrotic cells [9], [10]. As thymocytes undergo a high level of attrition due to selection events, such apoptotic thymocytes may present an abundant pool of self-proteins that potentially contribute to thymocyte selection.

Here, we analyze the role of CD205 in T cell development, including selection of both naturally diverse and MHC class I and class II restricted transgenic TCR repertoires. We demonstrate that in the absence of CD205, αβT cell development and selection proceeds normally, indicating that a deficiency in CD205-associated uptake of antigen within thymic microenvironments does not result in any gross effect on T cell selection.

## Materials and Methods

### Mice

CD205-deficient (*Ly75* ^−/−^) (Jackson Laboratories) [11], OT-I TCR transgenic [12], OT-II TCR transgenic [13] and SM1 TCR transgenic mice [14] were maintained within the Biomedical Services Unit at the University of Birmingham. All experiments were performed in accordance with UK Home Office regulations and were approved by the University of Birmingham Ethical Review Committee.

### Antibodies

Staining for flow cytometry was performed using the following antibodies: anti-AIRE Alexa Fluor 488 (5H12), anti-CD4 PECY7, eFluor 450 (GK1.5) or PerCPCy5.5 (RM4-3), anti-CD8a FITC, APC or eFluor 450 (53–6.7), anti-CD44 PECY7 (IM7), anti-CD69 FITC or PerCPCy5.5 (H1.2F3), anti-Ly51 PE (6C3), anti-EpCAM 647 (G8.8), anti-Foxp3 PE (FJK-16s), anti-TCRVα2 FITC (11-5812-82), anti-TCRβ APC eFluor 780 or PE (H57-597) anti-CD45 eFluor 450, PE-Cy7 or APC eFluor 780 (30F-11), CD45.2 FITC (104) (all EBioscience). Anti-IAb Pacific Blue (AF6-120.1), anti-CD25 APC (PC61), anti-CD62L APC (MEL-14) (All Biolegend). anti-H2kb biotin (AF6-88.5, BDPharmingen), followed by streptavidin PE-Cy7 (Ebioscience). TCR Vβ staining was performed using mouse TCR Vβ screening panel (BDPharmingen).

Staining for confocal microscopy was performed using the following antibodies: anti-Aire Alexa Fluor 488 (5H12), anti-CD4 (L3T4) conjugated to Alexa647, anti-CD8 biotin (CT-CD8β), anti-CD11c FITC (N418) (All Ebioscience), polyclonal rabbit IgG anti-β5t (MBL International), anti-medullary epithelium (monoclonal antibody, clone ER-TR5, kind gift of W. van Ewijk) [15]. For detection of ER-TR5 anti-rat IgM Alexa Fluor 594 was used. Streptavidin Alexa Fluor 555 was used to detect CD8 biotin. Detection of β5t Abs was achieved with anti-rabbit IgG Alexa Fluor 488 (all Invitrogen).

### Cell Isolation, Flow Cytometry and Cell Sorting

Isolation of thymic epithelial populations was performed as described [16]. Briefly, thymi were digested in RPMI (Hepes and L-Glutamine supplemented) (Sigma), 1 mM sodium pyruvate, 100 U/ml^−1^ penicillin, 100 mg/ml^−1^ streptomycin, 10 mM Hepes (all Gibco-Invitrogen), 5% fetal calf serum, 0.32 Wunsch U/ml^−1^ Liberase/thermolysin (Roche) and 50 Kunitz U/ml^−1^ DNaseI (Sigma). Thymi were digested for 40 minutes at 37°C. Digestion supernatant was collected and remaining tissue fragments subjected to a repeat digestion for a further 20 minutes. Supernatants were pooled and incubated with 5 mM EDTA for 5 minutes at 4°C. Thymic epithelial cells were enriched using CD45 immunomagnetic bead-mediated depletion (Miltenyi).

Acquisition of flow cytometry data was performed using a BD-LSR Fortessa cell analyzer and FACSDIVA 6.2 software (BD Biosciences, CA, USA). Flow cytometry data was analyzed using Flowjo software (Treestar). Cell sorting was performed using a Beckman Coulter XDP MoFlo (Beckman Coulter) and Summit software (Dako).

### Confocal Microscopy

Isolated tissues were embedded in OCT compound (Sakura Fintek UK) and frozen on dry ice. Frozen tissue sections were cut at 5 µm thickness, acetone fixed and stained with antibodies as detailed above. Confocal images were acquired using a LSM 510 Meta microscope (Zeiss) and analyzed using Zeiss LSM software. Quantitation of thymic regions was performed as previously described [17]. Briefly, cortical and medullary areas were identified via detection of β5t and ER-TR5 respectively. For each of two mice, five tissue sections at least 10 sections apart were analyzed. For each section, the total area of the tissue was imaged at ×100 final magnification and composite tilescan images were collated. The total area of cortical and medullary areas were automatically calculated using LSM Image Examiner software (Zeiss).

### Generation of Bone Marrow Chimeras

Recipient *Ly75* ^+/−^ and *Ly75* ^−/−^ mice were injected with red blood cell lyzed hematopoietic bone marrow cells freshly isolated from donor TCR transgenic mice (2×10^7^) one day following irradiation (900rad). Tissues were harvested for analysis 5 weeks after reconstitution.

### Statistical Analysis

Data were analyzed using the Mann Whitney non-parametrical statistical analysis (Graphpad Prism software). A *p*-value of <0.05 was considered significant.

## Results

### Normal Thymic Development and Organization in CD205-Deficient Mice

**Figure 1:**
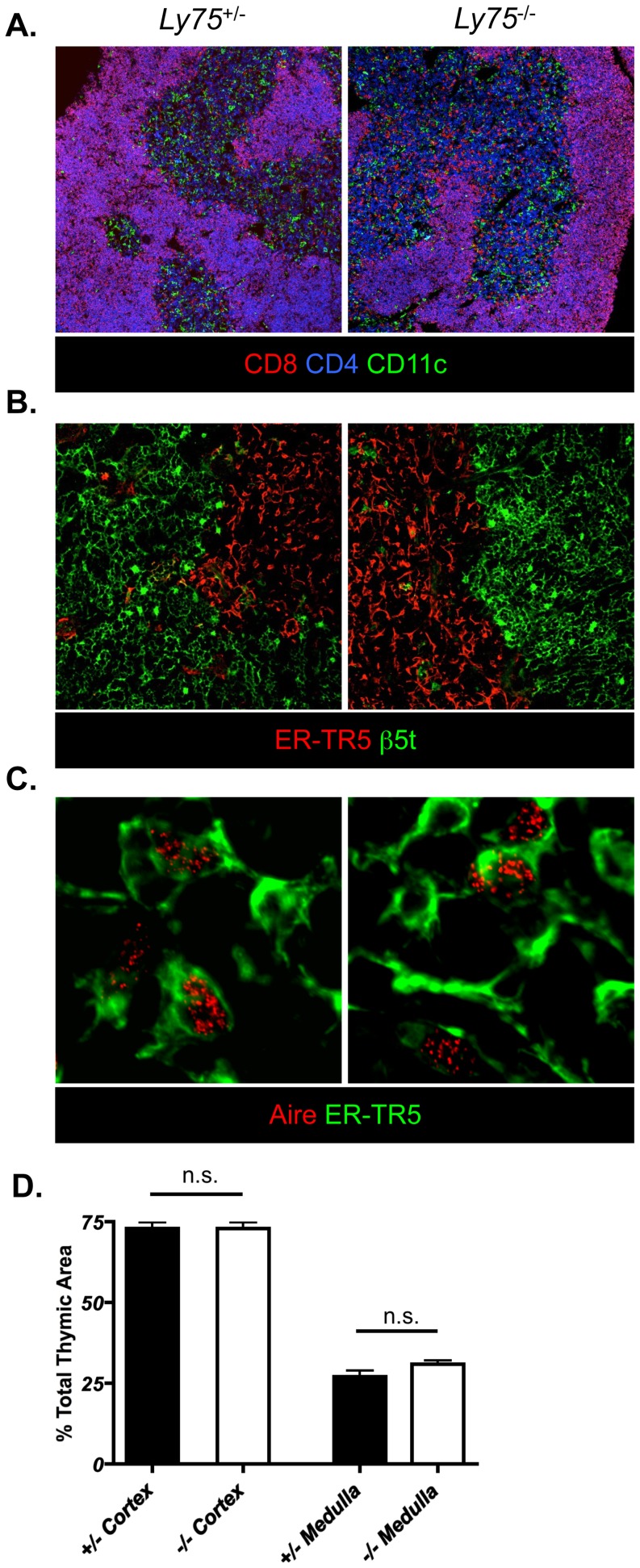
CD205-deficient mice demonstrate normal anatomical organization of thymic microenvironments. Frozen tissue sections of adult CD205-deficient (*Ly75* ^−/−^) and CD205-sufficient (*Ly75* ^+/−^) littermate control mice were analyzed by confocal microscopy for: (A) organization of thymocytes, defined by expression of CD4 (blue) and CD8 (red), and dendritic cells by CD11c (green), final magnification ×100, (B) thymic epithelium defined by β5t (cortex, green) and ER-TR5 (medulla, red), final magnification ×250, and the presence of Aire^+^ (red) ER-TR5^+^ MTEC (green), final magnification ×400 (C). Quantitative analysis of total cortical and medullary areas of CD205-sufficient (white bars) and CD205-deficient (grey bars) adult thymi (D). Data are representative of two individual experiments, n.s. = not significant.

Normal T cell development depends upon cortical and medullary thymic microenvironments. Amongst several cell surface molecules, CD205 positively discriminates cortical thymic epithelium. Furthermore, expression of CD205 within early embryonic thymus identifies cortical lineage-associated thymic epithelial precursors [18]. In order to exclude the possibility that CD205-deficiency leads to any perturbation in thymic epithelial development and/or organization of adult thymic epithelium, we compared both T cell and thymic epithelial compartmentalization in CD205-deficient (*Ly75* ^−/−^) mice by confocal microscopy (Figure 1). Analysis of thymocyte localization in CD205-deficient (*Ly75* ^−/−^) adult mice demonstrated a normal pattern of CD4 and CD8 distribution (Figure 1A, left panel) with CD4^+^8^+^ double positive (DP) thymocytes demonstrating cortical residency and post-positive selection CD4^+^8^−^ and CD4^−^8^+^ single positive (SP) thymocytes exhibiting medullary localization (Figure 1A). Analysis of CD11c^+^ dendritic cell positioning, essential for efficient negative selection and regulatory T cell development [19], was also found to be comparable in both CD205-deficient and -sufficient mice (Fig. 1A). Analysis of CTEC and MTEC segregation, defined by β5t and ER-TR5 respectively, demonstrated compartmentalization of differentiated CTEC and MTEC in CD205-deficient adult mice (Figure 1B). Aire positive MTEC, a subset of thymic epithelium essential for peripheral tissue antigen expression and deletion of auto-reactive T cell clones [20], were also present and distributed normally in mice lacking expression of CD205 (Figure 1C). The total area of cortical and medullary regions was calculated across multiple sections using composite tilescan images (Figure 1D). Both CD205-sufficient and -deficient mice demonstrated an equal contribution of cortical and medullary regions, together demonstrating that absence of CD205 expression within thymic tissue does not lead to either loss or alteration of thymic organization.

**Figure 2:**
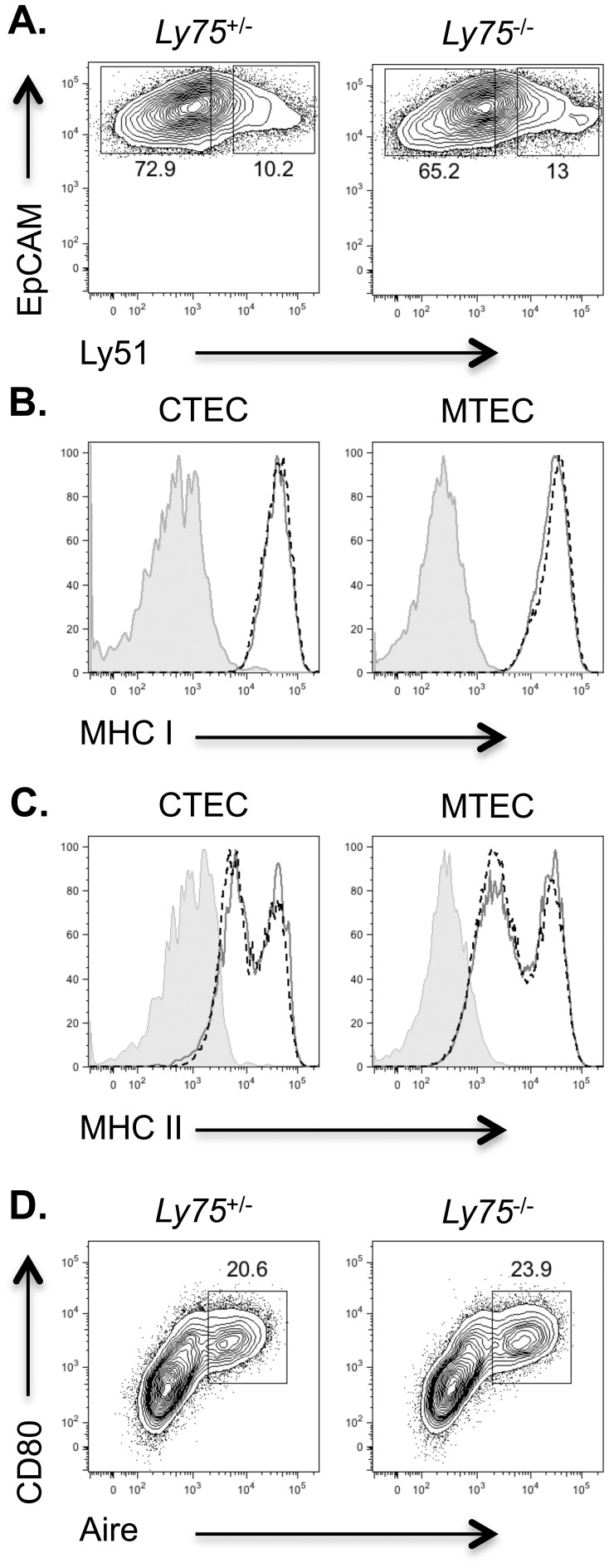
Flow cytometric analysis of CD205-deficient thymi does not reveal defects in thymic epithelial compartments. Thymi from adult *Ly75* ^+/−^ and *Ly75* ^−/−^ mice were enzymatically digested and analyzed by flow cytometry for: (A) CTEC (EpCAM^+^Ly51^hi^) and MTEC (EpCAM^+^Ly51^low^), cells gated on CD45^−^EpCAM^+^ thymic epithelium, (B) MHC class I and (C) MHC class II expression on CTEC and MTEC in *Ly75* ^+/−^ (grey solid line, open histogram) and *Ly75* ^−/−^ (black dashed line, open histogram) thymi, staining controls (filled histogram), and (D) Aire^+^ CD80^+^ MTEC, cells gated on CD45^−^EpCAM^+^Ly51^low^ cells. Data are representative of 3 separate experiments.

Thymic epithelial cells within the adult thymus exhibit heterogeneity, both between, and within CTEC and MTEC fractions [21], [22]. Cross-talk between developing thymocytes, and TEC plays a key role in regulating not only T cell development, but also that of thymic epithelial cells themselves [23], [24]. We therefore reasoned that if T cell development were disturbed in CD205-deficient mice, then this may subsequently feedback and impact upon thymic epithelial microenvironments. We therefore performed flow cytometric analysis of TEC compartments in adult thymus (Figure 2). The absence of CD205 in *Ly75* ^−/−^ mice precludes the use of this molecule to identify CTEC. We therefore used the cell surface marker Ly51 in order to identify Ly51^+^EpCAM^+^ CTEC and Ly51^−^EpCAM^+^ MTEC as previously described [25]. In agreement with confocal analysis, the presence of CTEC and MTEC fractions appeared to be normal within *Ly75* ^−/−^ mice at a per cell level (Figure 2A). Of note, mice lacking the CTEC specific MHC class II associated peptide processing Thymus-specific serine protease (Tssp) demonstrate reduced levels of MHC class II expression on CTEC [3], suggesting disrupted peptide processing may lead to alterations in MHC expression by TEC. We therefore analyzed MHC class I, and II expression in CTEC and MTEC of mice lacking CD205. Both MHC class I and II expression was found to be normal in the absence of CD205 (Figure 2B, C), indicating that both the maturation of TEC [26] and capacity to present self-peptides via MHC molecule expression were retained. Finally, analysis of Aire^+^ MTEC, a subset regulated by thymocyte cross-talk [27], demonstrated normal proportions in *Ly75* ^−/−^ thymus (Figure 2D). Together these data provide evidence that the absence of CD205 expression does not lead to any discernable alterations in the generation and/or maintenance of major thymic epithelial populations.

### Normal Selection of CD4 and CD8 T Cells Occurs in the Absence of CD205

**Figure 3:**
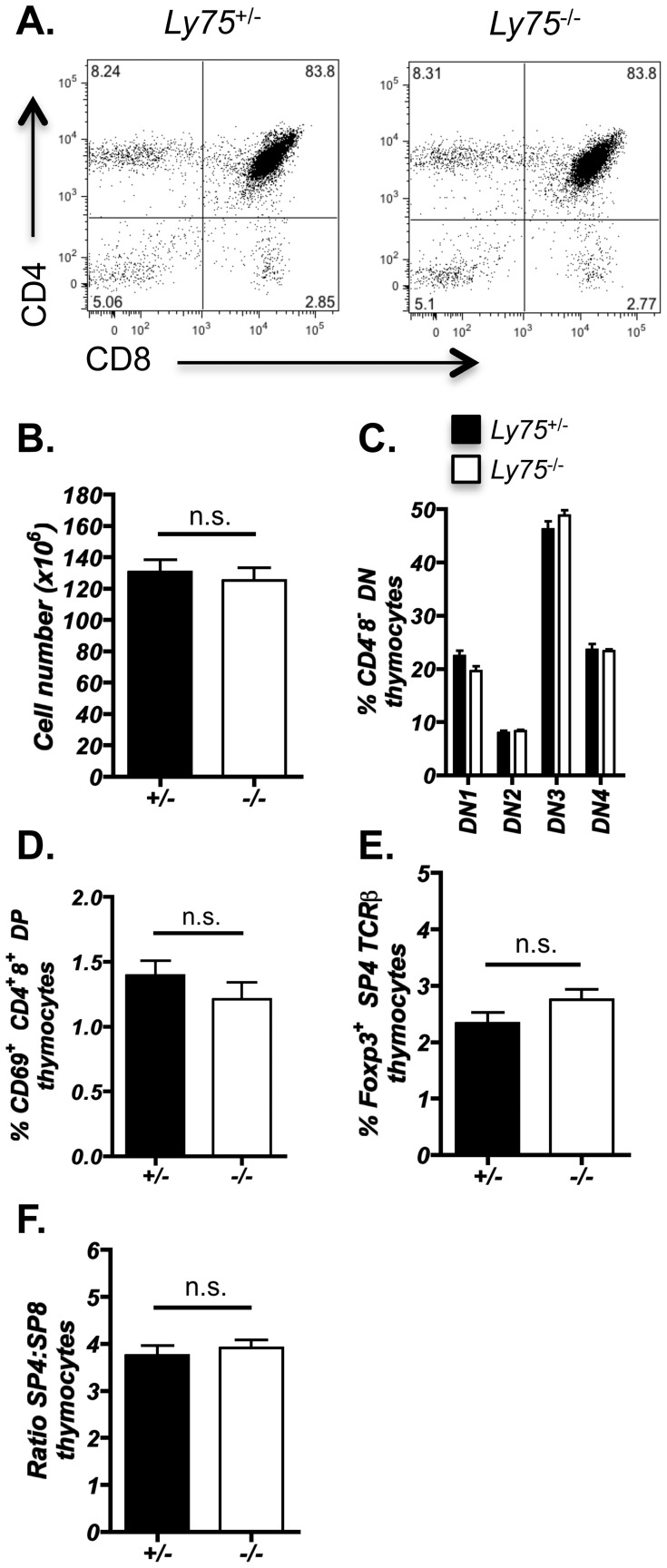
Efficient thymocyte development is unaffected in CD205-deficient thymus. Thymocytes isolated from adult *Ly75* ^−/−^ and *Ly75* ^+/−^ littermate controls were analyzed by flow cytometry for: (A) CD4 and CD8 distribution, representative of ≥10mice, (B) Total thymus cellularity, n = ≥10, (C) CD4^−^8^−^ double negative (DN) thymocyte distribution, CD44^+^25^−^ (DN1), CD44^+^25^+^ (DN2), CD44^−^25^+^ (DN3), CD44^−^25^−^ (DN4), n = ≥4, (D) CD69^+^ CD4^+^8^+^ double positive (DP) thymocytes, n = ≥4, (E) Foxp3^+^ T regulatory CD4 thymocytes, gated on CD4^+^8^−^ TCRβ^hi^ CD25^+^ Foxp3^+^, n = ≥7, and (F) CD4^+^8^−^ SP4 and CD4^−^8^+^ SP ratios, n = ≥4. Statistical analysis performed using Mann Whitney U test. n.s. (not significant). Error bars show ± standard error for indicated number (n) of mice examined.

**Figure 4:**
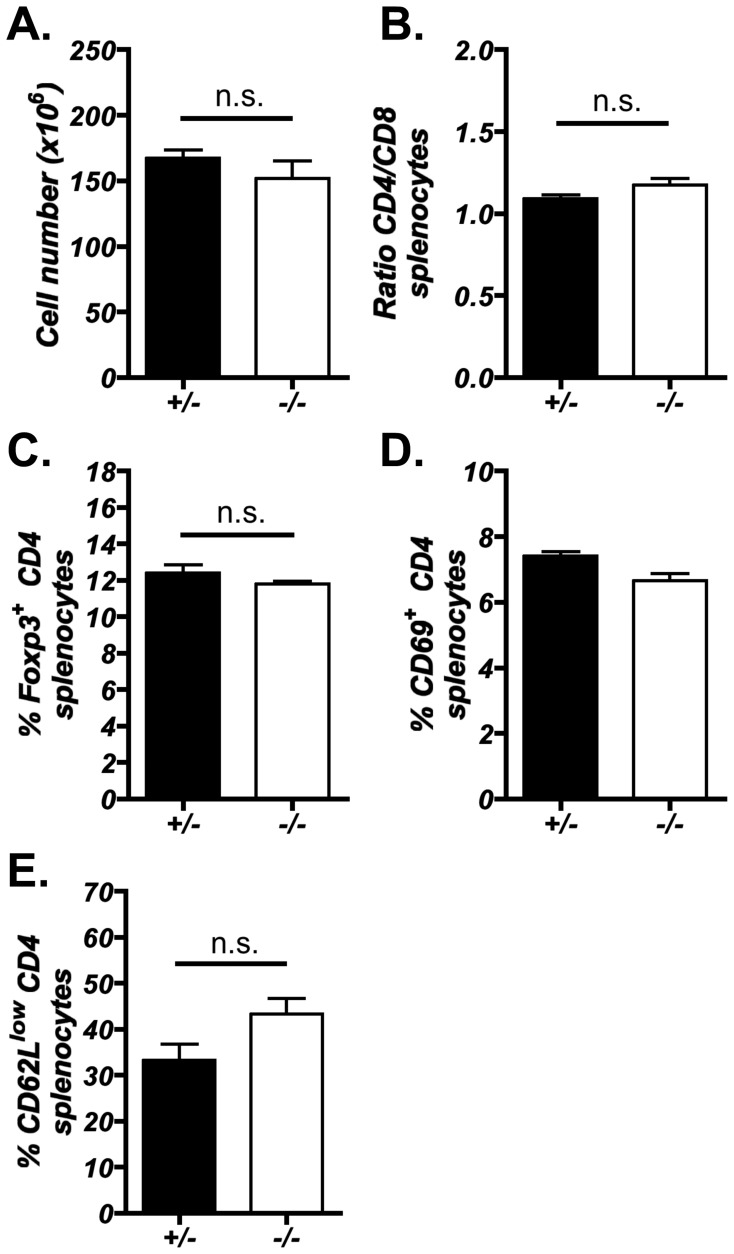
Peripheral T cells generated in the absence of CD205 appear normal and do not display signs of activation. Splenocytes isolated from *Ly75* ^−/−^ and *Ly75* ^+/−^ adult littermates were analyzed for: (A) total spleen cellularity, (B) CD4^+^8^−^ and CD4^−^8^+^ T cell ratios and (C) Foxp3^+^CD25^+^ CD4^+^8^−^ regulatory T cells. The occurrence of CD4^+^8^−^ splenocytes bearing an activated CD69^+^ and CD62L^low^ phenotype (D, E) was assessed in 10-week-old *Ly75* ^−/−^ and *Ly75* ^+/−^ mice. Error bars show ± standard error for n = ≥4 mice, n.s. (not significant) using Mann Whitney U test.

Given that previous investigations have proposed that CD205 may function in the uptake of self-proteins and contribute to the selection of T cells [6], [9], we next investigated the impact of CD205-deficiency on intrathymic αβT cell development. Flow cytometric analysis was performed on adult thymi from *Ly75* ^−/−^ mice (Figure 3). Total thymus cellularity was unaltered in *Ly75* ^−/−^ mice, as were the distribution of CD4 and CD8 expression defining the developmental progression of thymocytes (Figure 3A, B). Investigation of CD4^−^8^−^ thymocyte developmental subsets, as defined by CD25 and CD44, did not reveal any differences in *Ly75* ^−/−^ mice (Figure 3C). Analysis of CD69 expression by CD4^+^8^+^ double positive thymocytes, being indicative of recently positively selected thymocytes [28], [29], was also found to be unaltered in CD205-deficient mice (Figure 3D) suggesting that positive selection occurs at a similar frequency in CD4^+^8^+^ double positive thymocytes. In addition, comparison of natural CD4^+^Foxp3^+^ regulatory T cells, that develop in the thymus as a result of selection against self-antigens and are essential for suppression of autoimmune disease [30], revealed no significant difference between *Ly75* ^−/−^ and control mice (Figure 3E). Analysis of peripheral T cell compartments also revealed no alterations in either the total number of splenocytes, CD4 and CD8 T cells ratios or the presence of CD25^+^Foxp3^+^ CD4 regulatory T cells (Figure 4A, B, C), further indicating that T cell development proceeds normally in the absence of CD205. In addition, no significant increase in the presence of CD4^+^ T cells bearing an activated phenotype was observed in 10-week-old *Ly75* ^−/−^ mice (Figure 4D, E). Coupled with the observation that CD205-deficient mice lacked overt signs of autoimmunity and adoptive transfer of *Ly75* ^−/−^ splenocytes into Nude hosts did not lead to overt signs of autoimmune disease (data not shown), it is suggested that T cell generation in the absence of CD205 expression does not lead to a breakdown in central tolerance.

**Figure 5:**
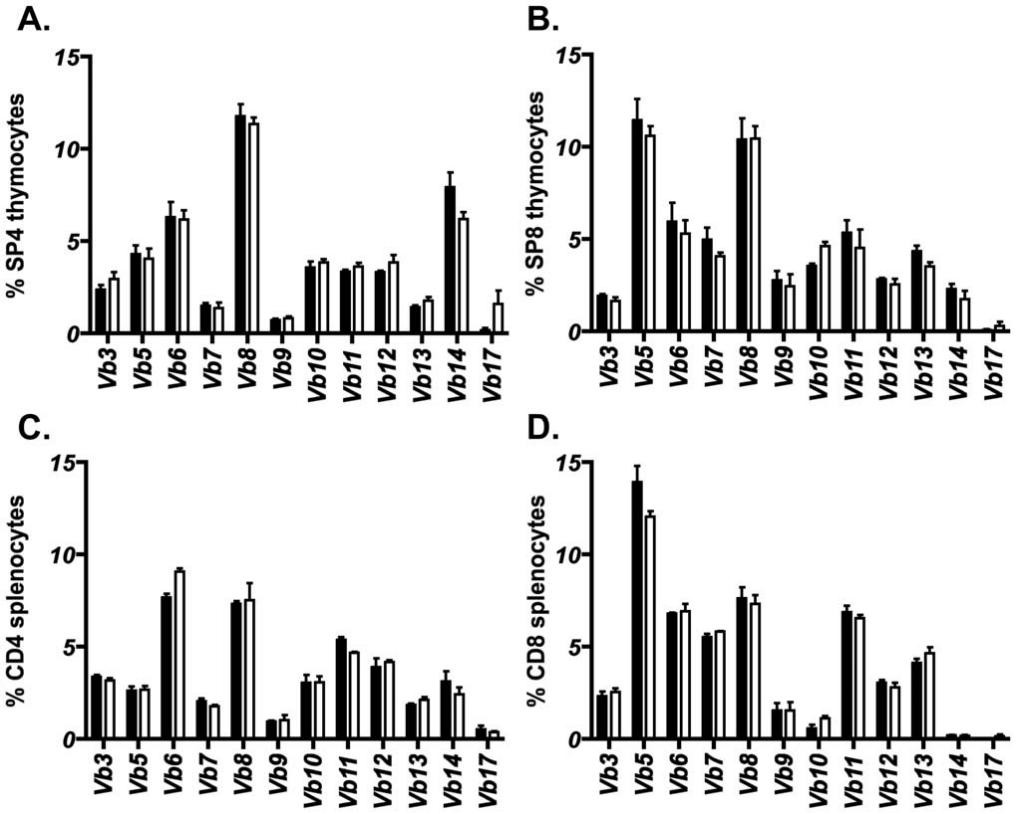
CD205-deficiency does not impact upon TCRVβ distribution in a polyclonal T cell repertoire. Flow cytometric analysis of TCRVβ distribution in adult *Ly75* ^−/−^ and *Ly75* ^+/−^ littermates for (A) CD4^+^8^−^ (SP4) thymocytes, (B) CD4^−^8^+^ (SP8) thymocytes, (C) CD4^+^8^−^ splenocytes, and (D) CD4^−^8^+^ splenocytes. Error bars show ± standard error for 3 independent experiments. Statistical analysis performed using Mann Whitney U test, no significant (n.s.) differences were determined between TCRVβ usage in *Ly75* ^−/−^ and *Ly75* ^+/−^ mice.

Altered thymic selection of developing thymocytes can potentially lead to disrupted CD4 SP and CD8 SP ratios and a shifted TCR repertoire [2], [4], [31]. The ratio of CD4 SP to CD8 SP thymocytes was found to be unaltered in *Ly75* ^−/−^ mice (Figure 3F), suggesting an absence of gross alteration to thymocyte selection. In order to investigate the T cell repertoire diversity that may become distorted in the presence of an altered selecting peptide array [32], [33], we analyzed the TCRVβ usage in both intra-thymic CD4 SP (Figure 5A) and CD8 SP thymocytes (Figure 5B). Within the polyclonal repertoire of unmanipulated *Ly75* ^+/−^ and *Ly75* ^−/−^ adult littermates, no significant differences were observed in the distribution of TCRVβ usage in either CD4 SP or CD8 SP thymocytes. Furthermore, analysis of TCRVβ usage within peripheral CD4 and CD8 splenic T cell compartments again did not reveal any significant differences between *Ly75* ^+/−^ and *Ly75* ^−/−^ adult mice (Figure 5C, D).

**Figure 6:**
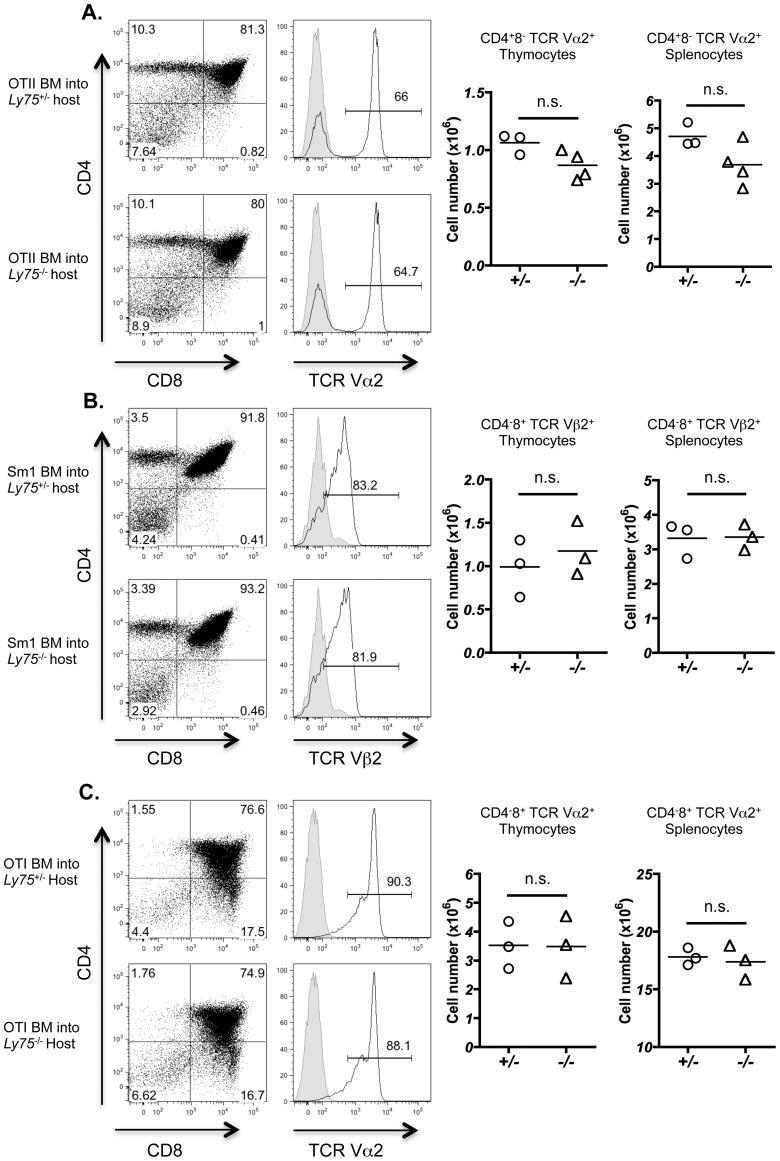
CD205 does not regulate positive selection of MHC class I and MHC class II restricted transgenic TCR specificities. Bone marrow (BM)-derived hematopoietic cells isolated from (A) OT-II and (B) SM1 MHC II-restricted, and (C) OT-I MHC I-restricted TCR-transgenic adult mice were transferred into lethally irradiated *Ly75* ^−/−^ or *Ly75* ^+/−^ littermate controls. Thymocytes were isolated 5 weeks after reconstitution and analyzed by flow cytometry for CD4 and CD8 (left hand panels). Histograms demonstrate TCR staining for transgenic-specific T cells, OT-II TCRVα2 (A), SM1 TCRVβ2 (B) and OT-I TCRVα2 (C) (open histograms), staining control (filled histogram). Right hand graphs demonstrate numbers of TCR transgenic T cells isolated from thymus and spleen, data points demonstrate total cell numbers for individual mice, gating as indicated (n = ≥3). Data analyzed using Mann Whitney U test. n.s. (not significant).

In order to determine subtle changes in TCRVβ use, we analyzed T cell development in *Ly75* ^−/−^ mice in the presence of T cells bearing a single fixed transgenic TCR. The absence of CD205 could lead to a slight, rather than drastic, drift in peptide presentation by CTEC that subsequently regulates thymocyte selection. As demonstrated by previous experiments, polyclonal T cell repertoires may be selected on limited arrays of peptides [34], therefore altered thymocyte selection could only become apparent in the setting of a single fixed monoclonal TCR repertoire. In order to investigate this possibility, *Ly75* ^−/−^ and *Ly75* ^+/−^ littermate controls were irradiated and reconstituted with hematopoietic bone marrow-derived cells isolated from TCR transgenic mice. Development of CD4 SP T cells was investigated using the MHC class II IA^b^ restricted TCR transgenic strains OT-II [13] and SM1 [14]. In both TCR transgenic specificities examined, no significant alteration was observed in CD4 SP selection as evidenced by either the proportion or total number of CD4 T cells generated in thymus or resident in spleen (Figure 6A, B). We next investigated whether CD8 T cell selection was influenced by potential CD205 contribution to peptide generation and T cell selection using MHC class I OT-I TCR transgenic cells restricted to H2-K^b^ [12]. Positive selection of OT-I CD8^+^ thymocytes was found to be comparable in both *Ly75* ^+/−^ and *Ly75* ^−/−^ host mice (Figure 6C). Taken together, these data suggest that expression of CD205 by CTEC does not significantly impact upon the development of T cells within the thymus, including positive selection of CD4^+^ and CD8^+^ thymocytes.

## Discussion

Within this study, we have assessed the functional role of the endocytic receptor CD205 in the regulation of thymocyte selection. We demonstrate that intra-thymic T cell development proceeds efficiently in the absence of CD205 expression in both a diverse polyclonal and single monoclonal T cell receptor restricted repertoire environment. Our data therefore suggest that binding and/or internalization of self-proteins via the CD205 pathway does not play an essential role in contributing to the array of self-peptides required for grossly normal thymocyte selection events.

CD205 acts as an endocytic C-type lectin-like molecule, being predominantly expressed by cortical thymic epithelium and dendritic cell subsets [6]. Whilst the exact nature of the ligands recognized by CD205 remain uncertain, previous data have demonstrated that CD205 acts as a recognition receptor for apoptotic cell uptake, with the proposed hypothesis that CTEC uptake and processing of dead thymocytes via CD205 may contribute to the pool of peptides responsible for driving thymic selection events and ultimately shaping the T cell repertoire [9], [10]. The functional role of CD205 in antigen uptake has primarily been investigated in the context of dendritic cell-mediated delivery of antigen to peripheral T cells. Protein delivered to dendritic cells via CD205-specific monoclonal antibody, leads to T cell tolerance via an induction of regulatory T cell generation, T cell anergy or deletion in the absence of dendritic cell activating factors [7], [35], or alternatively, T cell activation in the presence of dendritic cell stimulating signals [36]. It was therefore proposed that CD205 may play a potential role in thymocyte selection and central tolerance induction via uptake of self-protein in the form of apoptotic thymocytes and the subsequent processing and presentation of self-peptides by thymic epithelium [9]. In relation to a role for CD205 in central tolerance induction, it has previously been demonstrated that inefficient clearance of apoptotic cells may lead to a manifestation of autoimmune disease [37]. This may be of note, in that whilst CD205 mediated uptake of apoptotic thymocytes may potentially contribute to self-peptide generation and thymocyte selection, a secondary impact may occur via a role in clearance of excess apoptotic thymocytes and prevention of autoimmune disease induction. Our results imply that whilst CD205 demonstrates clear expression on thymic cellular compartments associated with normal T cell selection as previously described [6], absence of CD205 does not appear to significantly impact upon efficient thymocyte development. Importantly, neither CD4 nor CD8 T cell development was disrupted in either polyclonal or monoclonal transgenic TCR settings. This is of note as CD205-mediated uptake of antigen targets exogenous proteins not only to MHC class II pathways leading to presentation to CD4 T cells, but also presentation of antigen to CD8 T cells via cross-presentation into MHC class I pathways [7], [38].

The precise contribution of different sources of self-proteins for selecting self-peptides remains unknown. Whilst previous data have suggested an essential contribution of autophagy to thymocyte selection and tolerance-induction [4], opposing experimental models have contested that autophagy is not essential for the prevention of autoimmunity [5], possibly as a result of unaltered peptide presentation in the absence of autophagy pathway-derived peptides. However, it should be noted that the repertoire of T cell receptors in experiments describing an absence of autoimmunity in autophagy-deficient thymic epithelium have not been fully investigated.

Whether CTEC utilize internalization of exogenous proteins to generate self-peptide remains uncertain, however it has been demonstrated that CD205-specific monoclonal antibodies are efficiently internalized by cortical thymic epithelium [9]. The continued efficiency of T cell selection in CD205-deficient mice may either suggest redundancy between CD205 and other endocytic receptors potentially expressed by CTEC and/or thymic dendritic cells, or that endocytosis as a discrete mechanism does not play an essential role in the generation of peptides required for efficient thymocyte selection. Interestingly, previous studies have suggested that binding, and uptake of apoptotic thymocytes by a cortical thymic epithelial cell line, was not inhibited by CD205-IgG fusion proteins [9], potentially indicating functional redundancy in receptor-mediated binding and uptake of exogenous material by thymic epithelia. In summary, this study investigates a proposed role for CD205 in the efficient selection of developing T cells. Our findings demonstrate the lack of a major impact on thymocyte selection in CD205-deficient thymic microenvironments and suggest that CD205 is not essential for normal αβ T cell development.
